# Solution phase high repetition rate laser pump x-ray probe picosecond hard x-ray spectroscopy at the Stanford Synchrotron Radiation Lightsource

**DOI:** 10.1063/4.0000207

**Published:** 2023-10-27

**Authors:** Marco Reinhard, Dean Skoien, Jacob A. Spies, Angel T. Garcia-Esparza, Benjamin D. Matson, Jeff Corbett, Kai Tian, James Safranek, Eduardo Granados, Matthew Strader, Kelly J. Gaffney, Roberto Alonso-Mori, Thomas Kroll, Dimosthenis Sokaras

**Affiliations:** 1 SLAC National Accelerator Laboratory, Menlo Park, California 94025, USA; 2 Department of Chemistry and Energy Sciences Institute (ESI), Yale University, New Haven, Connecticut 06520-8107, USA

## Abstract

We present a dedicated end-station for solution phase high repetition rate (MHz) picosecond hard x-ray spectroscopy at beamline 15-2 of the Stanford Synchrotron Radiation Lightsource. A high-power ultrafast ytterbium-doped fiber laser is used to photoexcite the samples at a repetition rate of 640 kHz, while the data acquisition operates at the 1.28 MHz repetition rate of the storage ring recording data in an alternating on-off mode. The time-resolved x-ray measurements are enabled via gating the x-ray detectors with the 20 mA/70 ps camshaft bunch of SPEAR3, a mode available during the routine operations of the Stanford Synchrotron Radiation Lightsource. As a benchmark study, aiming to demonstrate the advantageous capabilities of this end-station, we have conducted picosecond Fe K-edge x-ray absorption spectroscopy on aqueous [Fe^II^(phen)_3_]^2+^, a prototypical spin crossover complex that undergoes light-induced excited spin state trapping forming an electronic excited state with a 0.6–0.7 ns lifetime. In addition, we report transient Fe Kβ main line and valence-to-core x-ray emission spectra, showing a unique detection sensitivity and an excellent agreement with model spectra and density functional theory calculations, respectively. Notably, the achieved signal-to-noise ratio, the overall performance, and the routine availability of the developed end-station have enabled a systematic time-resolved science program using the monochromatic beam at the Stanford Synchrotron Radiation Lightsource.

## INTRODUCTION

Pump probe studies using ultrashort laser pulses have greatly deepened our understanding of the fundamental processes underlying light-driven chemical reactions, biological functions, and material transformations.^1–4^ Specifically, the utilization of pump probe schemes with structural probes has revealed transient non-equilibrium structures with sub-Å spatial and femto- to picosecond temporal resolution. Ultrafast electron and x-ray diffraction methods have been widely used to study a variety of phenomena such as lattice dynamics associated with strain propagation, phase transitions, and melting dynamics in solids.^5–11^ In contrast, hard x-ray absorption spectroscopy (XAS) provides a local probe of electronic and nuclear structure around a specific element and is, therefore, particularly well suited to study molecular complexes in solution, where many chemical and biological reactions take place. XAS probes bound–bound transitions, bound-continuum transitions, and multiple scattering processes through the x-ray absorption near edge structure (XANES) in the proximity of the absorption edge, while the extended x-ray absorption fine structure (EXAFS) typically starts a few tens of eV above the absorption edge and can be used to extract nearest neighbor interatomic distances and coordination numbers of the absorbing element.^1,12^

During the past decade, optical pump hard x-ray probe experiments implemented at third-generation synchrotron radiation sources have greatly benefited from advances in high power, high repetition rate laser sources and the implementation of data collection strategies that can utilize the overall x-ray flux more effectively at the MHz revolution frequencies of the electron bunches.^13–18^ While the resulting increase by ∼3–4 orders of magnitude in the pump and probe repetition rates and, therefore, the usable x-ray flux has led to drastic signal-to-noise improvements, such measurements remain challenging for solid samples degrading under repeated laser irradiation at high repetition rates.^19,20^ Liquid samples, however, can be continuously replenished between successive laser pump and x-ray probe pulses with a rapid recirculating liquid jet sample delivery system. The time resolution of these experiments is limited to ∼100 ps by the electron bunch length unless operation modes or “time-slicing” schemes are invoked that can increase the time resolution at the expense of the usable x-ray flux.^21–24^ Nonetheless, these developments have unlocked high-quality picosecond XAS studies of dilute solutions or nanoparticulate suspensions, resolving subtle transient features in both XANES and EXAFS regions associated with electronic excited state dynamics, ligand photolysis kinetics in catalytic model systems and biomolecules, or charge carrier dynamics in nanomaterials relevant for solar energy conversion applications.^13,22,25–34^

In comparison, synchrotron-based picosecond hard x-ray emission spectroscopy (XES) studies remain less explored due to the lower x-ray photon count rates associated with the x-ray emission processes and the detection solid angle of the high-resolution x-ray spectrometers.^35–39^ XES is complementary to XAS and probes the local charge- and spin densities, and the ligand environment of 3d elements.^40–42^ Picosecond XES can be used to track photoinduced changes in spin and oxidation states at the metal sites via the more intense Kα (2p-1s) and Kβ (3p-1s) x-ray emission lines, while the valence-to-core (VtC) spectral range occurring ∼50 eV above the Kβ main line is more sensitive to the chemical environment. However, since the VtC lines exhibit 1–2 orders of magnitude lower intensity than the Kβ main lines, the application of synchrotron-based high repetition rate picosecond VtC XES remains particularly challenging.^35–37^

**FIG. 1.**
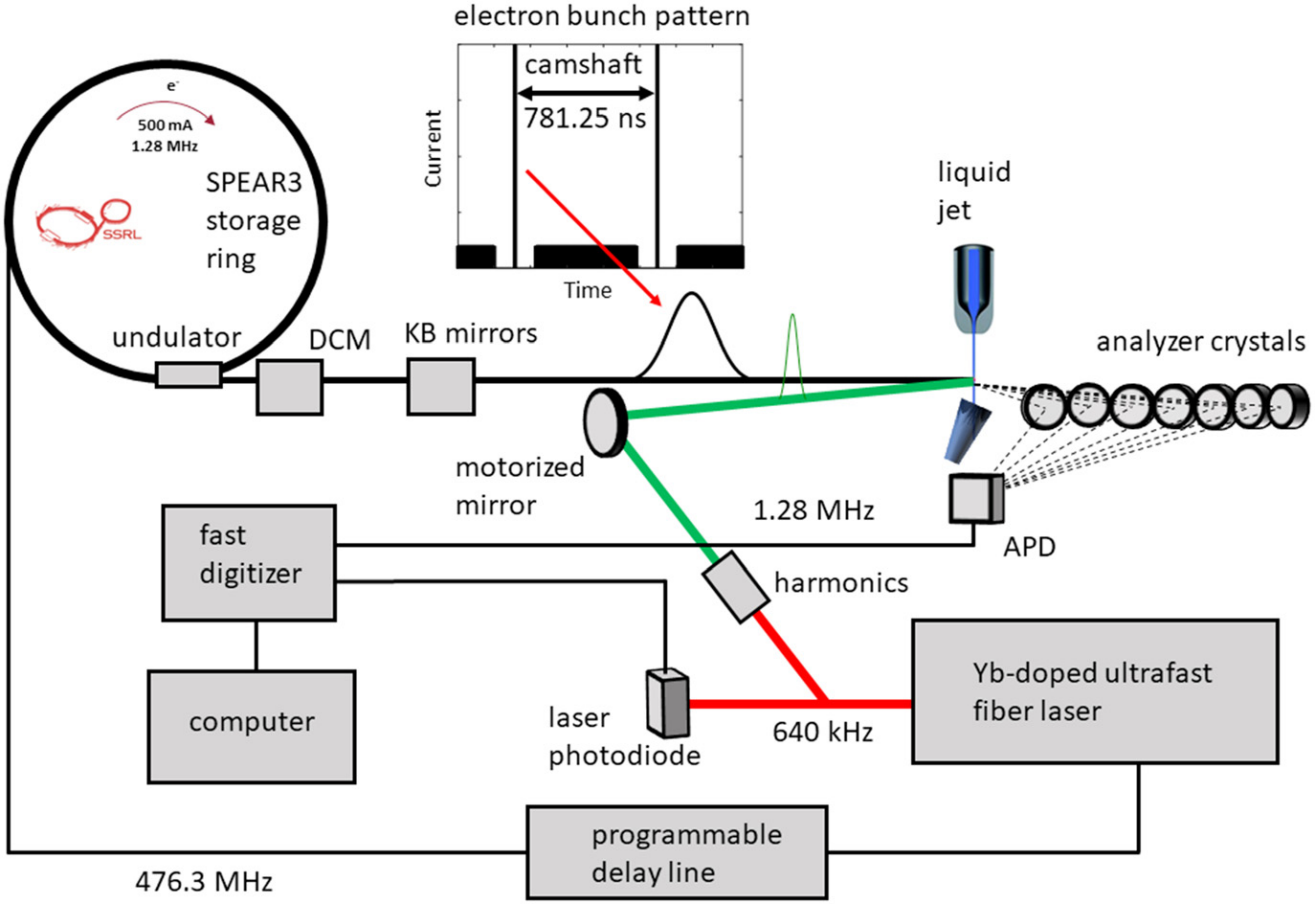
Schematic of the laser pump x-ray probe setup at SSRL beamline 15-2.

**FIG. 2.**
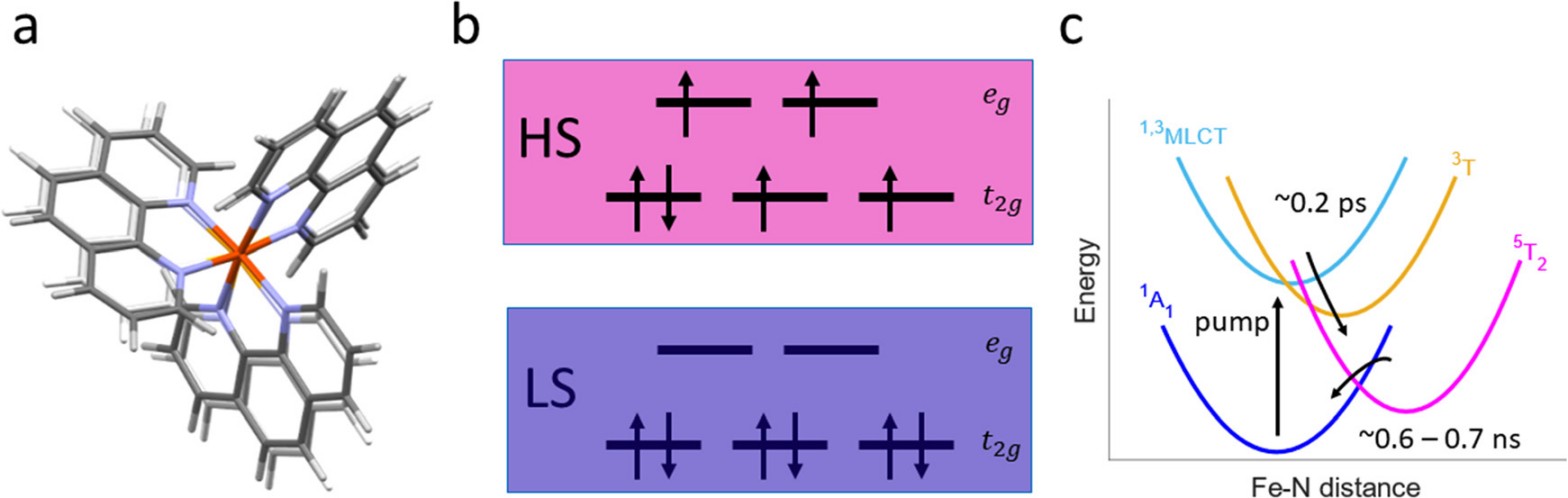
(a) Geometry optimized calculated structures of the low-spin (LS) and high-spin (HS) states of aqueous [Fe^II^(phen)_3_]^2+^. (b) LS and HS electronic configurations of the iron d-orbitals in octahedral symmetry. (c) Schematic of the photoinduced relaxation dynamics of aqueous [Fe^II^(phen)_3_]^2+^ as described in the main text.

Here, we present the implementation of a solution phase high repetition rate picosecond optical pump hard x-ray probe spectroscopy setup at beamline 15-2 of the Stanford Synchrotron Radiation Lightsource (SSRL) (Fig. 1). Notably, this capability is now available for routine time-resolved experiments, providing to the scientific community a reliable and systematic access for unfolding long-term research programs and accelerating scientific discovery. As a benchmark study, we have used an aqueous solution of 1,10-phenanthroline iron(II) sulfate, referred to herein as [Fe^II^(phen)_3_]^2+^, a complex that undergoes light-induced excited spin state trapping (Fig. 2).^15,16,43–47^ Upon visible excitation from the ^1^A_1g_ ground state into a singlet metal-to-ligand charge transfer (^1^MLCT) excited state (ES), the system is deactivated within ∼200 fs via successive intersystem crossing steps into a quintet metal-centered ES (^5^T_2g_). The ^5^T_2g_ state then relaxes back to the ^1^A_1g_ ground state within 0.6–0.7 ns in water.^15,47^

In the following, we describe in detail the experimental setup and then briefly discuss picosecond Fe K-edge XANES and EXAFS, and Fe Kβ main line and VtC XES results obtained for aqueous [Fe^II^(phen)_3_]^2+^.

## METHODS

### SPEAR3 storage ring and SSRL BL15–2

SPEAR3 is a low-emittance, high-brightness third-generation storage ring with a circumference of ∼234 m, a beam energy of 3 GeV, and an RF cavity frequency of 476.3 MHz. It provides a total current of ∼500 mA in top-off mode and can be operated with different electron bunch patterns. For pump probe measurements, a typical pattern contains four bunch trains (multi-bunch), where each individual bunch is filled with ∼1.8 mA (Fig. 1). The bunch trains are separated by three shorter gaps and a larger gap of ∼110 ns that contains a single electron bunch (“camshaft” bunch) that can be filled with close to the single bunch current limit and generates x-rays utilized as probe pulses. In addition to a single camshaft bunch, SPEAR3 has the capability to accommodate multiple camshaft bunches, which can be positioned either adjacent to each other or evenly distributed around the ring.

The single bunch current in a storage ring is constrained by the risk of longitudinal and transverse beam instabilities that emerge when trying to increase the charge density within a bunch. These instabilities are primarily a result of the interaction between the particle beam and the accelerator's components and structures, a phenomenon quantified in accelerator physics as beam impedance. As a result of its careful design to reduce the beam impedance, SPEAR3 has a high standard single bunch current limit of 22 mA in its nominal operating accelerator lattice configuration. Consequently, during time-resolved experiments, the camshaft bunch is typically loaded with 20 mA, leaving a comfortable margin below the limit. With adjustments in lattice chromaticity, this limit can be remarkably extended, allowing for the storage of up to 70 mA within a single bunch as demonstrated experimentally.^48^ Practical operational challenges, such as addressing elevated radiation loss with high single bunch current, must be addressed, but these open the door for brighter probe pulses.

To assess the bunch pattern and charge purity of the probe pulse, a time-correlated single photon counting system has been implemented.^49^ This system aids in identifying any stray charges in the vicinity of the camshaft bunch, which may result from injection timing jitter or stored bunch diffusion. While ongoing endeavors are dedicated to enhancing the probe pulse's purity by preventing the formation of these stray bunches, it is worth noting that they can always be effectively removed by the transverse multi-bunch feedback system.^50^ This feedback system possesses the capability to expel any single bunch from the accelerator ring without interruption of user operation as needed.

Furthermore, in order to alleviate the adverse effects associated with the presence of multi-bunch x-rays at the sample, which can potentially degrade samples and pose data readout challenges in sensitive experiments, efforts are under way for the implementation of the Pseudo Single Bunch (PSB)^51^ operation mode.

SSRL beamline 15-2 enables a range of different techniques, including XAS, XES, high energy resolution fluorescence detected (HERFD) XAS, resonant inelastic x-ray scattering, and x-ray Raman spectroscopy for studies in energy and material sciences, catalysis, or biochemistry. The beamline hosts an 87-period in-vacuum undulator (22 mm/period) and a liquid nitrogen cooled double crystal monochromator (DCM) utilizing sets of either Si(111) (∼3·10^13^ photons/s, ∼1 eV resolution at 7 keV) or Si(311) (∼4·10^12^ photons/s, ∼0.2 eV resolution at 7 keV) crystals. The monochromatic x-rays can be focused to a beam size of ∼5 *μ*m (V) × 35 *μ*m (H) with a KB mirror system consisting of horizontally and vertically deflecting, bendable x-ray mirrors. The accessible energy range of the beamline (∼4.8–18.2 keV using the KB mirrors) allows studies on most first-row transition metal K-edges and third-row transition metal L-edges. Within this energy range, the available x-ray flux varies by approximately one order of magnitude with lower fluxes occurring toward higher energies.

### Laser system and synchronization

**FIG. 3.**
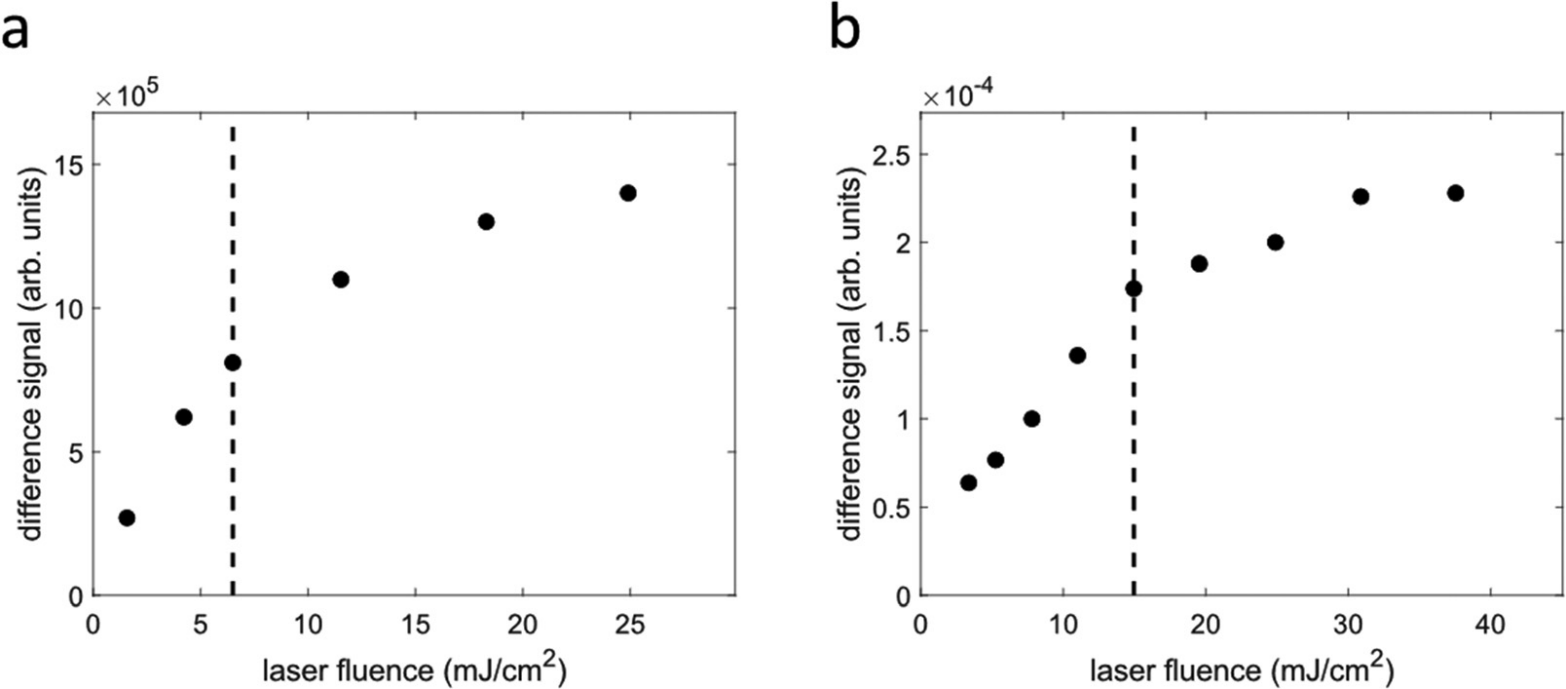
Fluence dependence of the photoinduced difference signals of aqueous [Fe^II^(phen)_3_]^2+^. Vertical dashed lines indicate the fluences used in the experiment. (a) X-ray absorption difference signal measured from a 2.5 mM aqueous solution at a pump probe delay of ∼100 ps with the incident energy fixed to ∼7125.6 eV. (b) X-ray emission difference signal measured from a 10 mM aqueous solution with the incident energy fixed to 8800 eV, the x-ray emission energy fixed to ∼7057.3 eV, and the pump probe delay set to ∼200 ps.

**FIG. 4.**
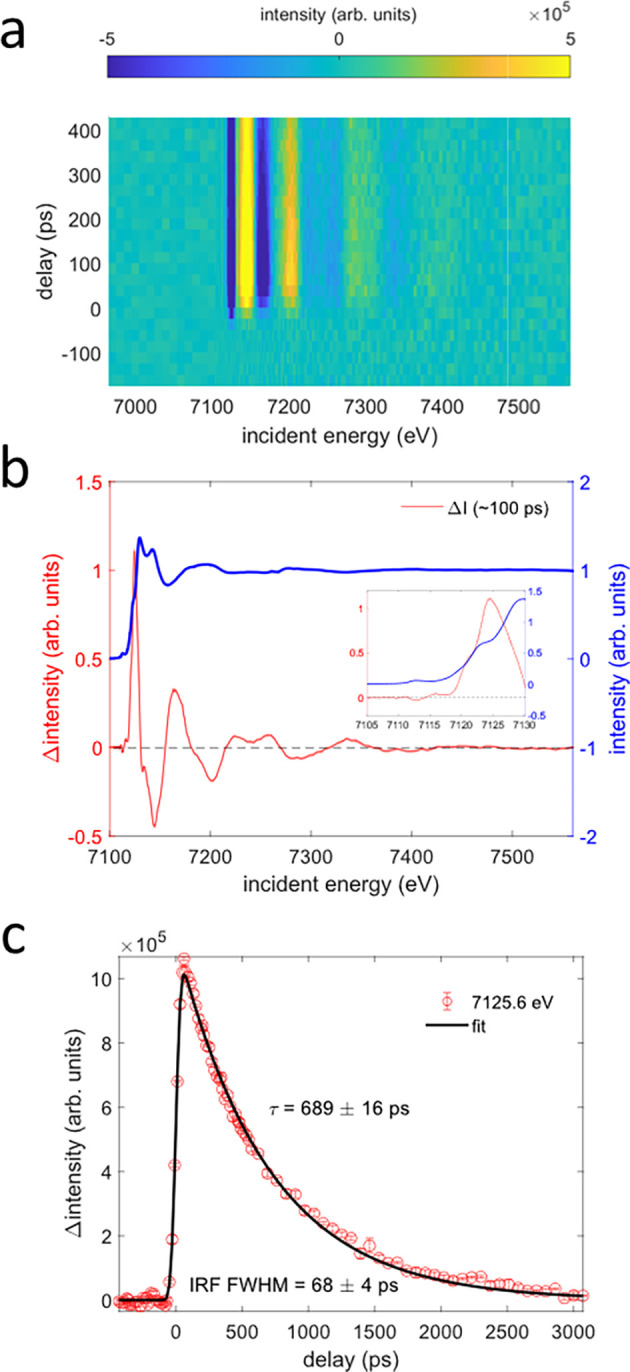
Picosecond Fe K-edge x-ray absorption spectroscopy of aqueous [Fe^II^(phen)_3_]^2+^. (a) Difference signal (pumped minus unpumped) as a function of pump probe delay and incident x-ray energy. (b) Difference signal (red) at a fixed pump probe delay of ∼100 ps. The inset highlights transient features in the pre-edge/edge region. The shaded area represents the standard error of all averaged scans. (c) Kinetic trace recorded at a fixed incident energy of ∼7126 eV shown together with a mono-exponential fit (R^2^ = 0.9989). Error bars represent the standard error of all averaged scans.

For optical excitation, we use a high-power (50 W) ultrafast ytterbium-doped fiber laser system from Amplitude Systemes (Tangerine). The laser system operates at a fundamental wavelength of 1030 nm and provides vertically polarized pulses with a duration of ∼300 fs (∼2.5 mm Gaussian beam diameter at the Tangerine output, M^2^ < 1.3). To generate an alternate series of pumped and unpumped x-ray probe measurements, the laser repetition rate is set to half the repetition rate (640 kHz) of the camshaft x-rays by using an external trigger signal derived from the 1.28 MHz electron bunch revolution frequency. To establish a stable pump probe delay, the Tangerine laser oscillator is phase-locked with the SPEAR3 RF signal at 476.3 MHz using commercial synchronization electronics integrated in the laser system (Amplitude Systemes Synchrolock). Synchronization is achieved through a built-in piezoelectric actuator, which continuously adjusts the laser oscillator cavity length to minimize the phase error between the oscillator and the SPEAR3 RF signal. To fine-tune the pump probe delay, we use a programmable delay line (Colby Instruments PDL-100A-40NS) that temporally shifts the 476.3 MHz RF signal before it enters the phase-lock loop. For coarse timing adjustments, a digital delay generator (Stanford Research Systems Model DG645) is used to shift the 640 kHz external trigger signal for the laser pulse picker in steps of ∼25 ns, corresponding to the laser oscillator round trip period. The 1030 nm output of the Tangerine laser is converted to 515 nm by using a 2 mm thick BBO crystal with an AR coating for type I second harmonic generation (Newlight Photonics Inc.). To separate the fundamental wavelength from the resulting second harmonic, a harmonic separator (R > 99.5% at 510–520 nm and T > 95% at 1020–1040 nm) is placed after the BBO crystal. Frequency conversion optics are also available to generate the third (343 nm) and fourth (258 nm) harmonics. When operating the laser at 640 kHz, typical achievable pulse energies are 78 *μ*J/26 *μ*J/5.4 *μ*J/3 *μ*J for 1030/515/343/258 nm, respectively. We then use a periscope in the laser beam path to match the x-ray and laser beam heights, focusing optics to increase the optical excitation fluence at the sample position and a mirror in a motorized mount (Zaber T-MM2-KT04U) to fine-tune the laser-x-ray spatial overlap. Typically, at the sample interaction point, the area of the focused laser spot is 1–2 orders of magnitude larger than for the x-rays, which ensures homogeneous sample excitation within the probed region and facilitates maintaining the spatial overlap during extended measurement periods. This becomes particularly relevant during transient EXAFS measurements (Fig. 4) to avoid distortions of the difference signal due to changes in spatial overlap arising from x-ray beam movements when scanning the incident x-ray energy across a wide range. For samples where an increase in the maximum achievable laser fluence is needed to achieve desired photoexcitation conditions, smaller laser spot sizes could be targeted by using beam expanders or focusing lenses with shorter focal lengths. However, for the measurements presented in this manuscript, this was not implemented because the available laser fluences were sufficient to well exceed the linear photoexcitation regime (Fig. 3).

### Sample delivery system

The sample delivery setup consists of a reservoir, an HPLC, gear, or peristaltic pump to recirculate the solution, a cylindrical jet nozzle, and a sample catcher that recovers and redirects the sample toward the reservoir. Through the nozzle, the recirculating sample forms a round liquid jet that is then pumped and probed in the interaction region a few millimeters below the nozzle exit, in a region of stable jet flow. The nozzle inner diameter can be chosen to optimize the optical and x-ray path lengths for given sample concentration and extinction coefficients. Generally, the rapid flow velocity allows continuously replenishing the measured sample to minimize effects due to laser and x-ray induced sample degradation. However, at the 1.28 MHz probe repetition rate, the jet flow velocities used in this study (see below) remain 1–2 orders of magnitude too slow to refresh the sample between subsequent x-ray probe pulses. Here, the measured difference signals remain unaffected because the photoexcited aqueous [Fe^II^(phen)_3_]^2+^ sample recovers completely between successive x-ray probe pulses. To extract reliable information for samples with photoproducts persisting beyond the ∼781 ns spacing between successive x-ray probe pulses, the influence on the observed difference signals could be minimized by lowering the pump and probe repetition rates, reducing the vertical optical pump size or using a faster liquid jet sample delivery system. The sample delivery chamber also has the flexibility to be isolated with Kapton windows providing air-tight conditions for air-sensitive molecules or for highly volatile solvents.

### Detectors and data acquisition system

For pump probe studies, we use avalanche photodiodes (APDs) with beryllium windows obtained from FMB Oxford (5 × 5 mm^2^, 110 *μ*m active silicon layer thickness), including pulse processing units (APD0002). The APDs are operated at ∼600 V, and we use the analog voltage signal output with a rise time of ∼5 ns, sufficient to isolate the camshaft from the multi-bunch x-rays. These voltage signals are then fed into a fast digitizer (Teledyne SP Devices ADQ14, 14-bit, 1 GSPS) that is triggered by the 1.28 MHz reference signal from SPEAR3. For each trigger, the raw voltage signal of the APDs is digitized in a 4–8 ns temporal window centered on the camshaft x-rays. To sort these x-ray intensities into pumped and unpumped datasets, a silicon photodiode with 1 ns rise time (Thorlabs DET10A2) was positioned on the laser table. The photodiode records the 640 kHz optical laser signal, which is then converted into a TTL signal with a digital delay generator (Stanford Research Systems Model DG645) and used by the digitizer to sort the recorded x-ray intensities into sets of pumped ( $I_{p}$) and unpumped ( $I_{u}$) x-ray intensities. The difference signal is then defined as $\Delta I=I_{p}-I_{u}$. For given incident x-ray energy or x-ray emission energy and pump probe delay, $I_{p}$, $I_{u}$, and ΔI are then averaged during a predefined period.

### Spatial and temporal overlap of the optical pump and x-ray probe beams

To spatially overlap the laser pump and x-ray probe beams, a ∼50 *μ*m tungsten pinhole (National Aperture, Inc.) is first centered in the focused x-ray beam by monitoring the transmitted x-ray intensity on a Si PIN photodiode (Hamamatsu S3204-09). The focused laser beam is then aligned through the pinhole using an optical mirror. To establish the temporal overlap between the two pulses, we use a fast photodiode (Hamamatsu G4176-05) with a bias-tee (Mini-Circuits ZX85-12G-S+) biased to 7 V. The photodiode is positioned at the spatial overlap position, and the signals of both laser and x-ray pulses are monitored on an oscilloscope (Teledyne LeCroy Wavemaster 816ZI-B, 16 GHz, and 80 GS/s), while the laser timing is shifted until both pulses overlap. The liquid sample jet is subsequently positioned in the spatial overlap region, and its position is finetuned to optimize the APD count rates. Once a pump probe difference signal is established on the sample, the spatial overlap can be further optimized using the motorized mirror mount used to steer the laser beam.

### Picosecond XAS measurements

To collect Fe K-edge XAS difference spectra and kinetic traces of aqueous [Fe^II^(phen)_3_]^2+^ in total fluorescence yield mode, we use the camshaft electron bunch with the Si(111) monochromator delivering ∼10^12^ photons/s at the sample position. The x-ray beam was focused to a FWHM of ∼8 × 37 ${\mu m}^{2}$, and the monochromator energy was calibrated by measuring an iron foil spectrum with a Si PIN photodiode (Hamamatsu S3204-09) and setting the first inflection point to 7112 eV. APDs were covered with Kapton tape and shielded on the sides with lead tape and then positioned as close as possible to the laser/x-ray interaction point, orthogonal to the incident x-ray beam. Using a 50 cm focusing lens, the 515 nm laser beam was focused to 175 × 210 ${\mu m}^{2}$ and spatially overlapped with the x-ray beam. We note that smaller spot sizes closer to ∼140 and ∼100 μm diameter are achieved with this configuration for the third (343 nm) and fourth (258 nm) harmonics, respectively. Based on a power titration curve measured at a pump probe delay of ∼100 ps and with the incident energy fixed to ∼7125.6 eV [Fig. 3(a)], the XAS measurement was performed with a pump fluence of ∼6.5 mJ/cm^2^. [Fe^II^(phen)_3_]^2+^ was dissolved in water at a ∼2.5 mM concentration and flowed through a 250 *μ*m inner diameter round nozzle forming a liquid jet. The sample was continuously recirculated at a flow rate of ∼6 ml/min using an HPLC pump (Shimadzu LC 20AD).

### Picosecond XES measurements

For XES measurements, the camshaft bunch was filled with 20 mA, and the x-ray beam was focused to ∼5 × 40 ${\mu m}^{2}$. To collect the x-ray emission from the sample, we used the SSRL multi-crystal Johann-type hard x-ray spectrometer,^52^ equipped with seven Ge(620) crystals with a 100 mm diameter placed on intersecting vertical Rowland circles of 1 m diameter. The spectrometer is scanned on the vertical plane to change the detected Bragg angle while maintaining the crystals on the Rowland circle trace. Generally, the beamline provides various crystals covering most fluorescence lines that lie in the accessible energy range. Although we do have a number of von Hamos XES instruments tailored for static measurements, here, we did not consider energy dispersive geometries for XES measurements because these require position sensitive detectors, which exhibit larger background to signal ratios and must function at MHz repetition rates. A polypropylene bag filled with helium was positioned between the crystals and the APD to minimize the signal attenuation and unwanted x-ray diffuse scattering contributions. The APD was covered with lead tape with a 1.5 mm slit, sealed with black Kapton tape, and positioned under a shielding box. The monochromator energy was first calibrated with an iron foil, and the x-ray emission energy was subsequently calibrated by measuring the elastic scattering peak position for various incident energies. The overall energy resolution for the XES measurements (including the Si(111) monochromator contribution) was estimated as ∼1.3 eV, based on the FWHM of the recorded elastic peaks. For XES measurements of such dilute samples, we expect to detect far less than one photon per camshaft pulse on average. Multi-photon events are, therefore, neglected, and a threshold is set in the digitizer firmware to discriminate between one- and zero-photon events. The digitizer counts one photon whenever any of the sampled data points exceeds a defined threshold. For the time scans, the incident x-ray energy was fixed to 8800 eV, and the x-ray emission signal was detected at 7056.4 eV. For the Kβ main line and VtC spectra, the incident energy was fixed to 8000 eV, and the pump probe delay was set to ∼100 ps. For photoexcitation, the laser was focused to a Gaussian size of 173 × 140 ${\mu m}^{2}$. The XES measurements were performed using an excitation fluence of ∼15 mJ/cm^2^, based on a power titration curve measured with an incident energy of 8800 eV, an x-ray emission energy of ∼7057.3 eV, and a pump probe delay of ∼200 ps [Fig. 3(b)]. [Fe^II^(phen)_3_]^2+^ was dissolved in water at ∼10 mM concentration and flowed through a 150 *μ*m inner diameter round nozzle. The sample was recirculated at flow rates in the range ∼2–8 ml/min using an HPLC pump (Shimadzu LC 20AD).

### Sample synthesis

To obtain a ∼2.5 mM aqueous solution of [Fe^II^(phen)_3_]^2+^, we have dissolved ∼70 mg of iron(II) sulfate heptahydrate (278.01 g/mol) and ∼135 mg of 1,10-phenanthroline, each separately in 50 ml de-ionized water. Both solutions were then mixed and stirred to obtain the final product. For ∼10 mM aqueous solutions, the amounts were scaled up accordingly. The estimated extinction coefficient at 515 nm is ∼10^4^ M^−1^ · cm^−1^.

## RESULTS AND DISCUSSION

Figure 4(a) shows the time-resolved Fe K-edge XAS difference signal ΔI of aqueous [Fe^II^(phen)_3_]^2+^ up to ∼0.4 ns. A singular value decomposition of this difference map reveals a single spectral component, as may be expected for the decay of the ^5^T_2g_ ES into the ^1^A_1g_ ground state. Figure 4(b), therefore, shows the difference signal recorded with improved statistics at a pump probe delay of ∼100 ps. The pre-edge region [Fig. 4(b), inset] shows a small increase in intensity near ∼7112 eV and an intensity decrease near 7113 eV, where the $1s-e_{g}$ pre-edge transition appears in the ground state spectrum. These transient features are consistent with the photoinduced change in electronic configuration from ${\left(t_{2g}\right)}^{6}{\left(e_{g}\right)}^{0}$ (^1^A_1g_) to ${\left(t_{2g}\right)}^{4}{\left(e_{g}\right)}^{2}$ (^5^T_2g_) [Fig. 2(c)] that creates two vacancies in the lower energy $t_{2g}$-subshell, giving rise to the positive feature around 7112 eV, while the $1s-e_{g}$ feature is reduced in intensity due to the transient occupation with two electrons. The recorded difference signal, therefore, reflects the population of the ^5^T_2g_ ES, which is formed within ∼200 fs. The overall shape of our Fe K-edge XAS difference signals is consistent with previously reported time-resolved XAS results for the compound.^15,16,44,45^ A combined structural analysis of the XANES and EXAFS regions up to k = 10 Å^−1^ has been reported by Zhan *et al.*^44^ For the ^5^T_2g_ ES, they found an increase in ∼0.17 Å in the Fe(II)–N bond lengths with respect to the ground state distance of 1.97 Å.

We have also recorded a kinetic trace with the monochromator energy fixed to ∼7126 eV [Fig. 4(c)]. A fit was then performed using the following fit equation for a mono-exponential decay:

$$I_{\mathrm{XAS}}\left(t\right)=I_{0}\cdot G\left(\sigma_{\mathrm{IRF}},t_{0},t\right)⊗\theta_{H}\left(t-t_{0}\right){\cdot e}^{-\frac{t-t_{0}}{\tau }}.$$ (1)

Here, G is a normalized Gaussian function, and $\theta_{H}$ is the Heaviside step function. Fit parameters are the amplitude $I_{0}$, the width $\sigma_{\mathrm{IRF}}$ of the instrument response function, the time zero position $t_{0}$, and the time constant τ. The fit ( $R^{2}\approx 1$) yields an IRF FWHM = 68 ± 4 ps ( ≈ 2.355 $\cdot \sigma_{\mathrm{IRF}}$), consistent with the expected temporal duration of the timing bunch. The fitted decay constant is obtained as τ = 689 ± 16 ps, in good agreement with previous measurements of the ^5^T_2g_ lifetime in aqueous solution.^15,45,47^

**FIG. 5.**
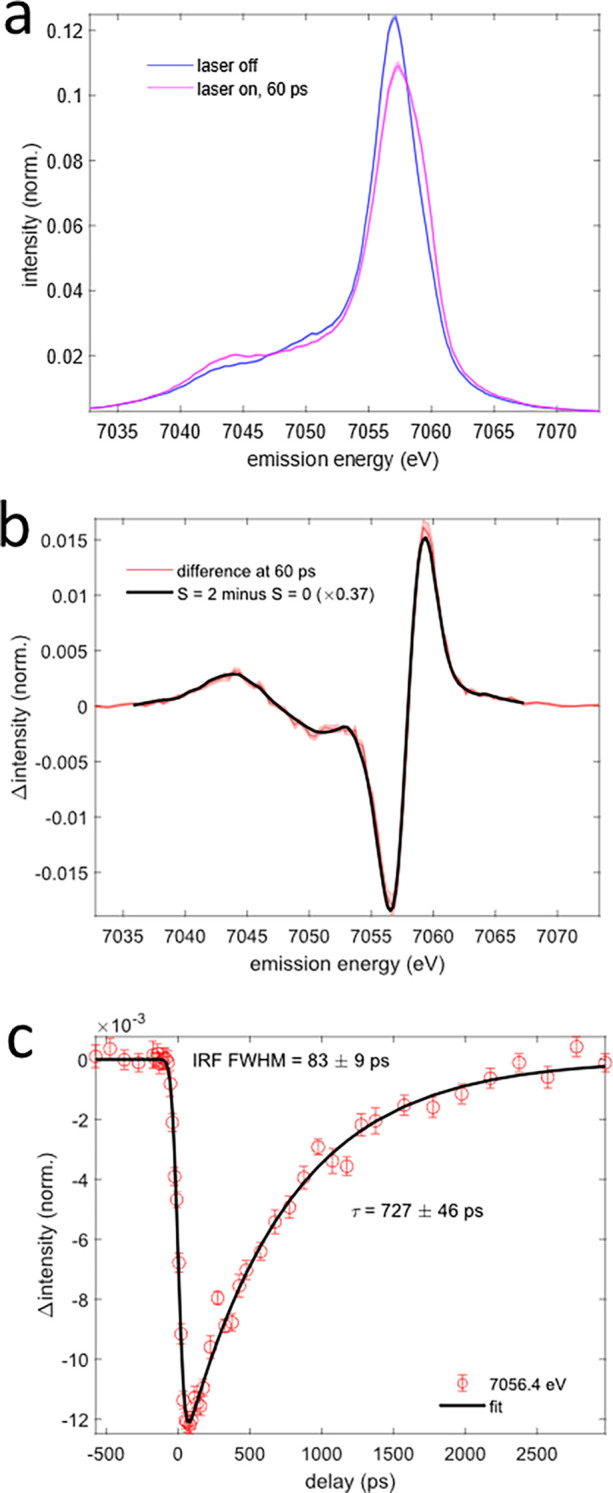
Picosecond Kβ main line x-ray emission spectroscopy of aqueous [Fe^II^(phen)_3_]^2+^. (a) Pumped (purple) and unpumped (blue) Kβ main line spectra recorded at a time delay of ∼60 ps. Shaded areas represent the standard error of all averaged scans. (b) Kβ main line difference spectrum compared with the scaled difference of an independently measured Fe(II) high-spin reference spectrum from Zhang *et al.*^57^ and the laser off spectrum [black line, Eq. (2)]. The shaded area represents the standard error of all averaged scans. (c) Kinetic trace of the Kβ main line difference signal recorded with the x-ray emission energy fixed to ∼7056 eV. Error bars represent the standard error of all averaged scans. The black line represents a fit using Eq. (1).

**FIG. 6.**
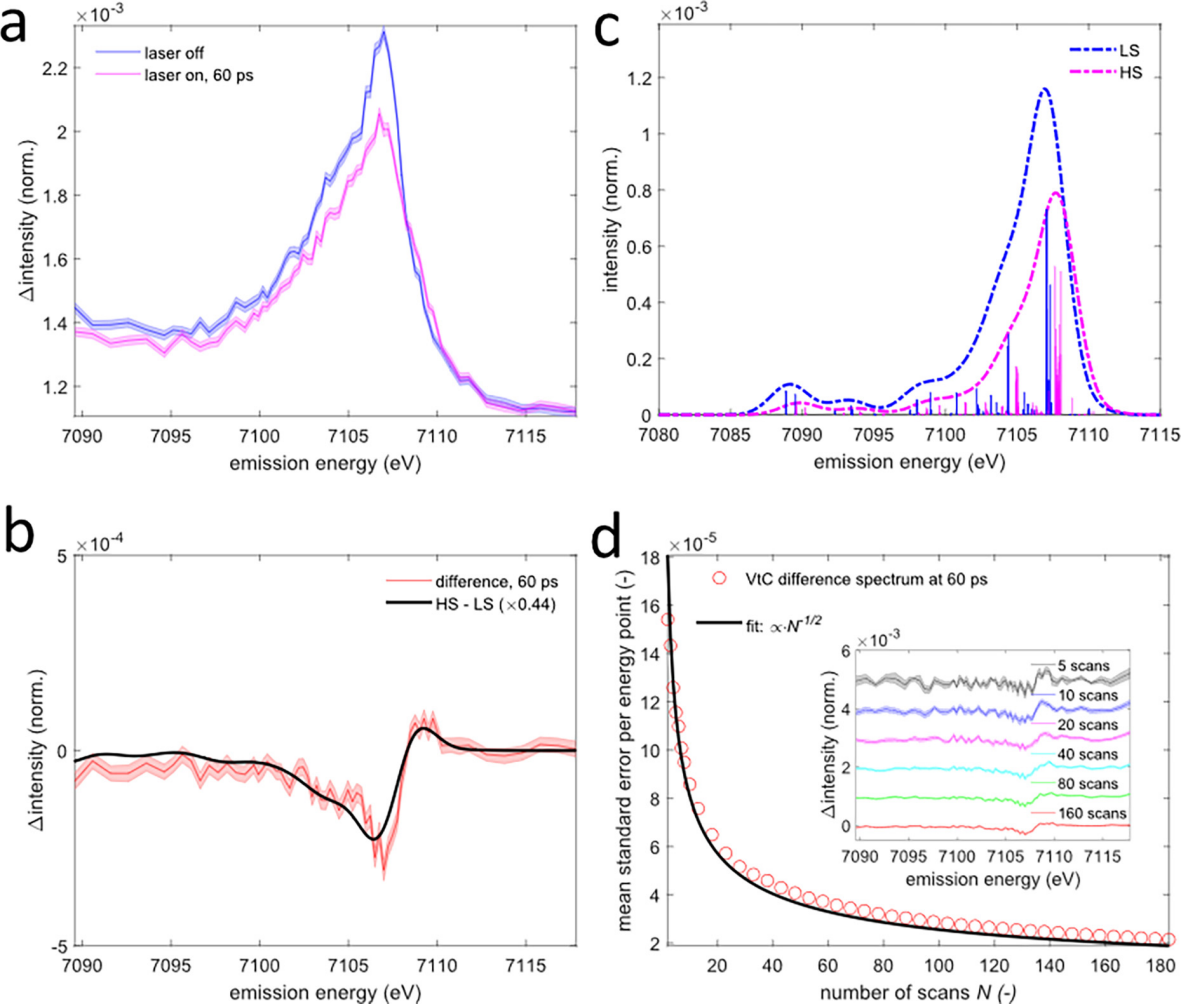
Picosecond valence-to-core x-ray emission spectroscopy of aqueous [Fe^II^(phen)_3_]^2+^. (a) Pumped (purple) and unpumped (blue) valence-to-core spectra recorded at a time delay of ∼60 ps. Shaded areas represent the standard error of all averaged scans. (b) Valence-to-core difference spectrum (red) compared with the scaled calculated high-spin minus low-spin difference spectrum (black line). The shaded area represents the standard error of all averaged scans. (c) Calculated low-spin (blue) and high-spin (purple) valence-to-core spectra. (d) Evolution of the mean standard error per energy point of the difference spectrum recorded at 60 ps with increasing measurement time (number of identical scans N). The black line represents a fit proportional to $N^{-1/2}$. The inset shows the mean difference spectrum after different numbers of recorded scans. The acquisition of a single scan containing 66 energy points takes ∼5.6 min.

Figures 5(a), 5(b), 6(a), and 6(b) show the Fe Kβ main line and VtC x-ray emission difference signals of aqueous [Fe^II^(phen)_3_]^2+^ recorded at ∼60 ps. The Fe Kβ main line signal stems from the 3p-1s x-ray emission process. The line shape is sensitive to the exchange interaction between the Fe 3d electrons and the 3p hole, metal-ligand covalency, and ligand field splitting effects.^53–56^ On the femtosecond timescales, ultrafast Fe Kβ main line spectroscopy has frequently been utilized to track metal spin and oxidation state changes during photoinduced processes in molecular solutions.^56–63^ Figure 5(b) also shows a fit of the Fe Kβ main line difference signal $\Delta I_{K\beta }$ at ∼100 ps using

$$\Delta I_{K\beta }\left(E\right)=\gamma \cdot \left(I_{K\beta }^{HS}\left(E\right)-I_{K\beta }^{LS}\left(E\right)\right),$$ (2)

$\text{where}\;I_{K\beta }^{LS}$ is the measured low-spin (LS) [Fe^II^(phen)_3_]^2+^ laser off spectrum, $I_{K\beta }^{HS}$ is an independently measured and properly normalized Fe(II) high-spin (HS) reference spectrum ([Fe(phenanthroline)_2_(NCS)_2_]) from Zhang *et al.*,^57^ and γ≈0.37 is the photoexcited fraction of HS molecules at 100 ps. The good agreement between the HS model and the experimental difference spectrum confirms the population of the ^5^T_2g_ MC ES and suggests the absence of additional states with different spin multiplicity on this timescale. A fit of the kinetic trace recorded at a fixed x-ray emission energy of 7057 keV using Eq. (1) yields an IRF FWHM of 83 ± 9 ps and decay constant of τ = 732 ± 46 ps. While the decay constant agrees with that obtained from the XAS measurement, the fitted IRF FWHM for the XES measurement is somewhat longer. However, this result is consistent with the higher camshaft bunch current used in the XES measurement, resulting in slightly longer camshaft x-ray pulses.^64^

The VtC x-ray emission lines occur at higher x-ray emission energies and exhibit 1–2 orders of magnitude lower intensities than the main lines. Therefore, these x-ray emission lines have remained more challenging to measure with femto- or picosecond techniques.^36,37,56^ Here, we report the collection of transient VtC spectra at ∼60 ps [Figs. 6(a) and 6(b)]. The standard error calculated from the scan-to-scan variation and averaged over all measured x-ray emission energy points of the difference spectrum is shown in Fig. 6(d) as a function of the number of identical data collection scans N. As demonstrated by the fit shown in Fig. 6(d), the standard error approximately decreases with $N^{-1/2}$, which suggests that the measurement is nearly counting statistics noise limited. The recorded VtC difference spectrum [Fig. 6(b)] is negative throughout most of the VtC-range consistent with an overall reduction in metal-ligand orbital overlap associated with the Fe(II)-N bond weakening and elongation in the ^5^T_2g_ state.^65^ Around 7109–7110 eV, the difference spectrum shows a small positive feature. For a more quantitative analysis, we have conducted density functional theory calculations of the VtC x-ray emission spectra for aqueous [Fe^II^(phen)_3_]^2+^ [Fig. 6(c)]. These calculations were performed for the lowest energy LS and HS states using the ORCA 4.2.1 package.^66^ Geometry optimizations were carried out using the B3LYP functional, def2-TZVP basis set^67^ and DFT-D3 dispersion correction.^68^ Solvent effects were included using the conductor-like polarizable continuum model (CPCM) for water.^69^ VtC x-ray emission spectra were calculated using the B3LYP functional. The ZORA-def2-TZVP basis set was used for all elements except for the Fe atom, where the CP(PPP) basis set was used with a special integration accuracy of 7.^35,37,65,70^ Scalar relativistic effects were considered via the zero-order regular approximation (ZORA).^71^ Only dipole transitions were included in the spectra. The calculated transitions were broadened using a 3.0 eV FWHM Gaussian function and shifted by 22.4864 eV to overlap the peaks of the calculated and experimental ground state spectra. After geometry optimization, the six Fe(II)–N bond lengths are ∼2.00 Å in the LS ground state. In the HS state, four of the six Fe(II)–N bonds elongate to ∼2.18 Å, while the two axial bonds elongate to ∼2.20 Å, consistent with slight axial distortion. While the calculated absolute bond lengths for the LS ground state are somewhat higher than reported in the EXAFS study by Zhan *et al.*,^44^ the Fe–N bond elongation of ∼0.18 Å is in good agreement. The calculated spectra for these structures are shown in Fig. 6(c), and their scaled difference is compared with the experimentally measured difference spectrum at ∼60 ps in Fig. 6(b). The shape of the calculated difference spectrum agrees well with the experimental difference and captures the small positive feature on the high energy side associated with a slight shift of the HS state transitions toward higher energies.

To independently estimate the HS population fraction from the VtC analysis, we have rescaled the calculated LS and HS VtC spectra such that the calculated LS ground state spectrum agrees with the normalized and background subtracted experimental ground state spectrum. The background of the experimental VtC spectrum was subtracted by using the tail of a Kβ main line fit determined with a pseudo-Voigt function including a constant offset. The calculated difference spectrum is then fitted to the experimental difference using a scaling factor $\gamma_{\mathrm{VtC}}$. The best fit is achieved for $\gamma_{\mathrm{VtC}}\approx 0.44$, which is not too different from the HS fraction γ≈0.37 estimated from the Kβ main line analysis. Again, the overall good agreement between the calculated HS-LS difference and the experimental VtC difference spectrum suggests negligible contributions of additional transient species to the observed difference signal, consistent with a large quantum yield for the formation of the HS state.

## CONCLUSIONS

We have reported the implementation of solution phase high repetition rate laser pump hard x-ray probe capabilities at the SSRL. As a benchmark study, we have performed comprehensive picosecond hard XAS and XES measurements for an Fe-based model complex in aqueous solution that undergoes light-induced excited spin state trapping into a HS state with a lifetime of 0.6–0.7 ns. The transient XAS results including both XANES and EXAFS regions resolved detailed photoinduced electronic and structural features associated with the change between the LS and HS electronic configurations at the metal site. The picosecond Fe Kβ main line XES measurement directly confirms the increase in the metal spin state due to the LS-HS transition. Density functional theory calculations of the transient VtC x-ray emission spectra support an average metal-ligand bond expansion of the Fe(II)-N distances by ∼0.18 Å.

The achieved sensitivity in the XAS experiment will enable challenging studies on solution phase samples with low solute concentrations and poor stability under measurement conditions such as, for instance, hydrogenase active site model systems. As recently demonstrated with femtosecond resolution at the Linac Coherent Light Source, combining both the nuclear structure sensitivity of VtC XES with the spin and oxidation state sensitivity of Kβ main line XES can be a powerful probe of complex electronic excited state or chemical reaction dynamics.^56^ We expect this capability could be of particular interest to identify long-lived charge transfer excited states in earth-abundant metal-based photosensitizer candidate compounds, which are being intensively studied for solar energy conversion applications.^72–74^

## Data Availability

The data that support the findings of this study are available from the corresponding authors upon reasonable request.
